# Evaluation of urine albumin-to-creatinine ratio analysis using strip test as a screening method for urinary albumin determination in primary care

**DOI:** 10.1515/almed-2024-0068

**Published:** 2025-05-01

**Authors:** Paula Liébanas García, Silvia Montolio Breva, Teresa Sans Mateu

**Affiliations:** 16455 Laboratori Clínic ICS Camp de Tarragona – Terres de l’Ebre – Hospital Tortosa Verge de la Cinta, Tarragona, Spain

**Keywords:** albuminuria, screening test, reagent strips, chronic kidney disease

## Abstract

**Objectives:**

The determination of urinary albumin, crucial for chronic kidney disease (CKD) diagnosis and monitoring, typically employs quantitative techniques as the gold standard. However, semi-quantitative methods such as urine reagent strips show promise as cost-effective and rapid screening tools. This study aims to evaluate the feasibility of utilizing Meditape UC-11A strips (Sysmex, Kobe, Japan) for albuminuria screening compared to quantitative assays measuring urine albumin-to-creatinine ratio (ACR).

**Methods:**

We systematically analyzed 1,627 strip samples using Meditape UC-11A strips whenever quantitative urinary albumin measurement was required. We compared the ACR measured by strip test with the gold standard (quantitative method determined by Atellica, Siemens, Marburg, Germany) and calculated diagnostic indicators. Additionally, we assessed the economic implications based on test prices (€2.88 for quantitative albumin plus creatinine, and €0.93 for a Meditape UC-11A strip).

**Results:**

Receiver operator characteristic (ROC) curve analysis was conducted to determine the optimal cut-off point. The concordance rate achieved using the calculated cut-off point was 90.78 %. Using the quantitative test as the gold standard, the sensitivity, specificity, positive predictive value (PPV), and negative predictive value (NPV) of ACR, analyzed using Meditape UC-11A, were 84.2 , 91.9, 62.7, and 97.3 %, respectively. Our findings suggest a potential saving of 81.2 % on quantitative tests during the study period, amounting to €2,291.65.

**Conclusions:**

This study supports the use of Meditape UC-11A strips for detecting abnormal levels of albuminuria, thus offering a viable alternative to quantitative measurement methods. Substantial cost savings can be achieved through this approach.

## Introduction

Chronic kidneydisease (CKD) is characterized by a gradual loss of kidney function and it is becoming a growing global public health issue. Clinical practice guidelines have been implemented to improve CKD outcomes [1], [2].

CKD is defined as the presence of abnormalities in kidney structure or function persisting for more than 3 months [3]. This includes: 1) glomerular filtration rate (GFR) less than 60 mL/min/1.73 m^2^; 2) albuminuria; 3) abnormalities in urine sediment, histology, or imaging suggestive of kidney damage; 4) renal tubular disorders; or 5) history of kidney transplantation.

CKD is categorized into five stages according to the GFR. However, due to uncertainty regarding threshold values for GFR, some meta-analysis were performed [4]. Based on these analyses, staging by the level of albuminuria was added in addition to the level of GFR, and GFR stage 3 was subdivided into 3a and 3b to better reflect prognosis. An association has been observed between staging and cardiovascular risk: in ended-stages, the risk of developing cardiovascular diseases is higher [5].

Albumin, the most abundant protein in urine in healthy patients, is the most used marker of kidney damage and CKD [6]. Albuminuria can predict the development of kidney damage in the earliest stages of the disease and is capable, by itself, of causing tubulointerstitial damage through the activation of proinflammatory mediators [7].

The albumin determination has some advantages over the determination of total proteins in urine: it not only presents more accuracy and precision but also, it is a better marker of protein loss due to an alteration in glomerular filtration [8].

The gold standard to detect protein loss through the kidney is a quantitative test in a sample from the 24-h urine collection. However, this measurement correlates very well with the albumin-to-creatinine ratio (ACR) measured in urine obtained by first-morning spot samples and solves several drawbacks: ACR facilitates sample collection and reduces the variance due to both the patient’s hydration status and the circadian rhythm.

ACR level considered normal is less than 30 mg/g, if it is between 30 and 300 mg/g it is considered moderate albuminuria and values ​​higher than 300 mg/g high albuminuria [1].

Although quantitative methods are considered as the gold standard, albuminuria, creatinuria and therefore ACR can also be easily detected by semiquantitative techniques such as the test strip.

Lately, advancements in technology have led to notable strides in automated urinalysis. The adoption of complementary metal oxide semiconductor (CMOS) technology has boosted analytical sensitivity and holds potential for microalbuminuria testing [9], [10]. Moreover, both microscopy-based urine particle analysis and its alternative, flow cytometry, have seen significant progressions.

Several studies have demonstrated the effectiveness of these techniques of semi-quantification [11], [12], [13], [14] which would serve as a screening tool. Compared to quantitative methods, these are faster and cheaper but have the defect of being less sensitive.

People suffering from diabetes and hypertension are known to have an increased risk of CKD. In 2014, 8.5 % of the population over 18 years suffered from diabetes and it is expected to increase [15], [16] On the other hand, more than a quarter of the adult population was estimated to have hypertension in 2000, but it is projected to increase by 60 % by 2025 [17], [18].

According to the guidelines [19], populations at an increased risk for CKD should be screened at least annually to detect patients in early stages of CKD and thus prevent its progression and the appearance of cardiovascular damage [20], [21].

Given that CKD and its risk factor diseases are expected to increase in the next few years and taking into account that albumin tests are currently highly requested and used in very different patient populations, it is necessary to find a screening method which should be cheap, fast and with adequate sensitivity to deal with this increasing workload and to reduce cost and time effort.

The working hypothesis is that ACR analysis using reagent strips could be used as a semiquantitative screening test in urine samples from Primary Care (PC) patients, thus avoiding quantitative tests, saving resources and shortening results response times when the result is negative.

The main aim of the study is to evaluate the feasibility of using reagent strips for the semi-quantification of ACR as a screening test in PC samples. The cost-benefit of this new strategy is also described, comparing the use of reagent strips with the quantification of albumin and creatinine in urine.

## Materials and methods

### Setting and patients

The urine samples were processed in the clinical laboratory located in Hospital de Tortosa Verge de la Cinta (HTVC). It is an acute hospital that belongs to the Catalan Health Service and covers 179.891 patients.

Although the clinical laboratory receives samples from inpatients and outpatients, only samples from adult PC patients were used in this study.

### Study design

A retrospective and unicentric cross-sectional study was carried out at HTVC during the months of October and November 2023. This study was approved by IISPV (Institut d’Investigació Sanitària Pere Virgili) Ethical Committee.

### Data collection

Spontaneous urine samples were collected in preservative-free containers using standard hospital procedure and were processed the same morning in the HTVC laboratory.

### Laboratory methods

Quantification of albumin was measured according to the immunoturbidimetric assay enhanced with PEG (Atellica, Siemens, Marburg, Germany). The sample containing albumin is diluted and subsequently reacted with specific antiserum to form a precipitate that can be measured by turbidimetry at 340/596 nm.

Creatinine quantification is measured according to the multi-enzyme assay based on the Suzuki and Yoshida method (Atellica, Siemens, Marburg, Germany). At the end of these reactions, a colorimetric compound is generated whose absorbance can be measured at 545/694 nm.

The analysis using the Meditape UC-11A reagent strips was carried out with the UC-3500 equipment (Sysmex, Kobe, Japan). The strips have three pads: one detection pad for creatinine based on the Benedict–Behre method (with a measuring range of 10–300 mg/dL) and two measuring pads for albumin based on the principle of protein error of tetrabromophenol blue, a pH indicator. The difference between the two pads is the amount of tetrabromophenol blue, the first contains 10 μg of tetrabromophenol blue (with an albumin range up to 0.150 g/L) and the second 5 μg of tetrabromophenol blue for concentrations of albumin above 10 g/L. Albumin concentration is measured as 10, 30, 80, 150 or>150 mg/dL and creatinine as 10, 50, 100, 200, or 300 mg/dL. Before starting the study, within and between-run precisions was evaluated with control material, UC-Control High Level and UC-Control Low Level (Sysmex, Kobe, Japan) to ensure internal quality control.

The ACR values were obtained by dividing the albumin concentration by the creatinine concentration for both the Atellica assay and the test strip method. The data that support the findings of this study are available from the corresponding author upon reasonable request.

### Cross-sectional study

In this retrospective study we compared quantitative ACR measured on a chemistry platform (Siemens, Marburg, Germany) with a semiquantitative test performed on an automated system based on dipstick evaluations (Sysmex, Kobe, Japan) using the first-morning samples of 1,627 patients.

To carry out this study, the semi-quantitative ACR was systematically measured in all PC patients who had a quantitative urine albumin determination requested. Each sample was analyzed with the semi-quantitative method using the UC-3500 equipment and later quantitatively with the Atellica autoanalyzer (Siemens, Marburg, Germany).

Data was collected during the months of October and November 2023. We export from the equipment, the quantitative albumin and creatinine values ​​as well as their ratio and the three test strip results (ACR, albumin and creatinine) to Microsoft Excel file.

### Statistical analyses

Sensitivity, specificity, positive predictive value (PPV) and negative predictive value (NPV) were calculated for the strip tests. Receiver operator characteristic (ROC) curves were performed using R packages pROC [22] to determine the optimal cut-off point [23], as demonstrated in previous studies [24], [25].

To identify the most effective threshold, we calculated Youden’s index (J). This index is a commonly used metric for evaluating diagnostic tests, as it maximizes the balance between sensitivity and specificity, thereby identifying the cut-off point that provides the best overall diagnostic performance.

A Kolmogorov-Smirnov test for normality was performed to determine whether the data followed a normal distribution. Based on the results, a Spearman’s rank correlation coefficient was used to evaluate association between ACR values obtained from the strip test and the quantitative method [26]. Additionally, we conducted a chi-square test to compare the frequency of positive and negative ACR results between the two measurement methods, considering quantitative ACR as the gold standard and values<30 mg/g as negative.

Statistical significance was set at a p-value<0.05, and all analyses were conducted using R Commander.

For the cost-benefit analysis, we estimated potential savings by comparing the cost of quantitative albumin and creatinine measurements (€2.88) with that of the Meditape UC-11A strip (€0.93).

## Results

A total of 1,627 participants, comprising 49 % females and 51 % males, with a mean age of 66 years (with a range from 15 to 99 years) were enrolled in the study. All samples underwent assessment of the ACR using both reagent strips and the quantitative method as the reference standard. The Meditape UC-11A strip test, performed with UC-3500, demonstrated satisfactory precision in terms of both intra- and inter-assay variability when tested on control material.

Table 1 presents the distribution of albumin and creatinine levels in 1,627 samples analyzed using the Meditape UC-11A assessments. We can observe that albumin levels are typically underestimated when assessed with the Meditape-UC11A, while creatinine levels are often overestimated, especially at higher concentrations.

**Table 1:** Distribution of albumin and creatinine according to Meditape UC-11A strip test assessments.

	Meditape UC-11A	Quantitative assay
	n	Median (range)
Albumin, mg/L		29.3 (3–2,160.3)
10	1,312	5 (3–45.2)
30	168	28.1 (3.1–75.7)
80	66	67.7 (14.2–134)
105	2	215 (203.9–226.8)
150	79	398 (8.4–2,160.3)
Creatinine, mg/dL		97.3 (7.9–452.6)
10	83	24.4 (7.9–78.2)
50	543	52.1 (27.5–125.4)
100	556	94.9 (60.4–226)
200	374	156.5 (97.8–304.2)
300	71	235.4 (155.7–452.6)

**Figure 1:**
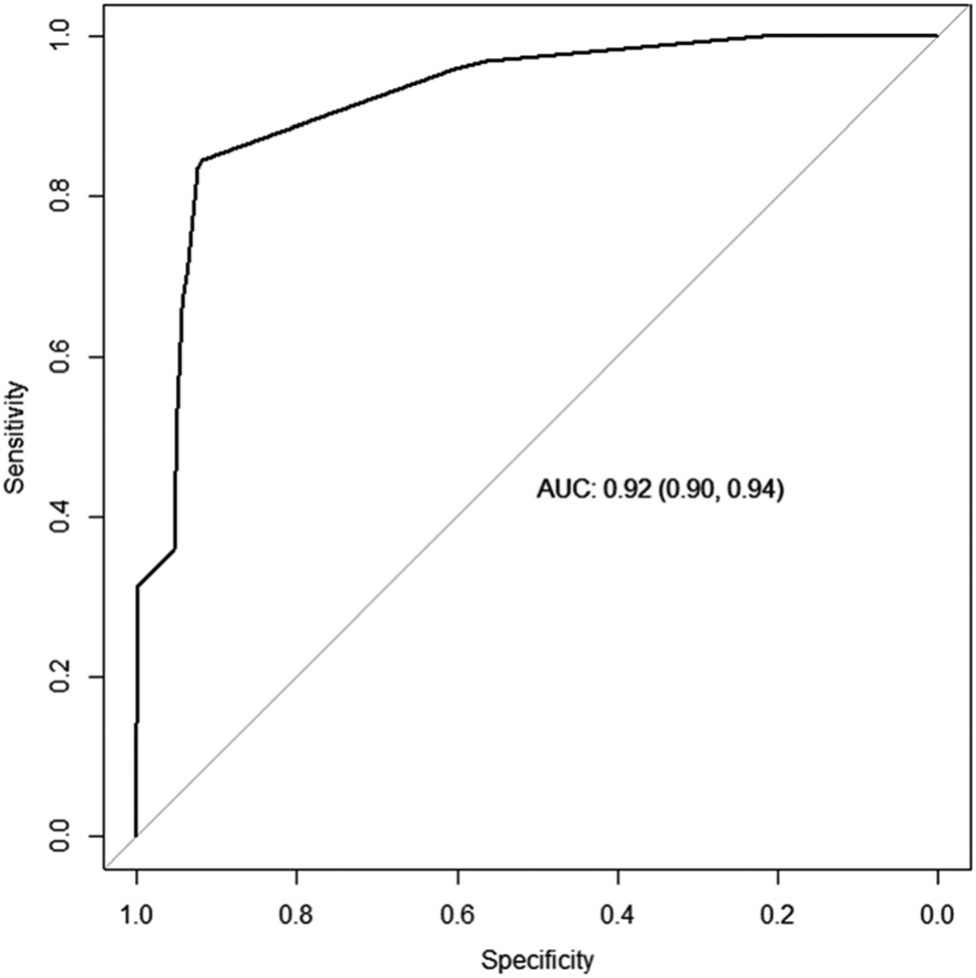
ROC curve obtained for ACR measurement using Meditape UC-11A test.

To assess the diagnostic accuracy of the ACR measurements obtained by reagent strips, we constructed a ROC curve, which yielded an Area Under the Curve (AUC) of 0.92 (95 % CI: 0.90–0.94), indicating excellent discriminative ability in detecting ACR abnormalities (Figure 1).

The optimal cut-off point for ACR measurement using reagent strips was determined to be 26.67 mg/g, with a sensitivity of 84.2 % and specificity of 91.9 %. This cut-off point was identified as the one that maximized Youden’s index (J=0.76), confirming its robustness in optimizing test performance (Supplementary Table S1).

Considering the ROC curve analysis, Table 2 presents a comparison of the frequencies of positive and negative ACR results from 1,627 samples analyzed using the strip test with those obtained using the quantitative gold standard assay. Out of the total samples analyzed with the Meditape UC-11A, 306 (18.81 %) exhibited an ACR of ≥26.67 mg/g, while 1,321 (81.19 %) showed<26.67 mg/g. In contrast, the quantitative assay identified 228 (14.01 %) samples with an ACR of ≥30 mg/g and 1,399 (85.99 %) with<30 mg/g. The chi-squared analysis revealed a strong association (χ^2^=742.76, p-value<2.2×10^−16^), suggesting that results from both methods are closely aligned. The diagnostic accuracy metrics, including sensitivity, specificity, PPV and NPV for the UC-3500, are detailed in Table 3.

**Table 2:** Comparison of ACR using the Meditape UC-11A strip test with those analyzed using the quantitative assay.

Meditape UC-11A (Sysmex)	Atellica, siemens	
	≥30 mg/g	<30 mg/g
≥26.67 mg/g	192	114
<26.67 mg/g	36	1,285

**Table 3:** Sensitivity, specificity, PPV and NPV of ACR analyzed by UC-3500, using the cut-off of 26.67 mg/g.

	ACR Meditape UC-11A
	(95 % CI)
Sensitivity, %	84.2 (78.9–88.4)
Specificity, %	91.9 (90,3–93.2)
PPV, %	62.7 (57.2–68)
NPV, %	97.3 (96.3–98)

We further assessed the relationship between ACR values obtained using the strip test and those from the quantitative method. Due to the non-normal distribution of the data demonstrated by Kolmogorov–Smirnov test, we employed Spearman’s rank correlation, which indicated a significant and robust positive correlation between the two methods (Spearman’s rho=0.82, p-value<2.2×10^−16^). This high level of agreement confirms the reliability of the strip test as a screening tool, showing that it aligns well with quantitative measurements and can effectively support the detection of urinary albumin levels in PC patients.

False-negative ACR results were observed via the strip test in 36 cases (2.21 %) with levels ranging from 30.16 to 116.18 mg/g (median: 46.47) among the 1,627 samples. False-positive ACR results were seen in 114 (7.01 %) cases with levels ranging from 2.4 to 29.10 mg/g (median: 8.16).

Overall, 1,321 (81.2 %) urinary albumin quantitative tests could have potentially been avoided, resulting in hypothetical savings of €2,291.65 during the study period. If this strategy had been routinely applied over a one-year period, the savings would have been €20,601.72.

## Discussion

Our study evaluated the efficacy of Meditape UC-11A strips as a screening method for detecting albuminuria in PC patients. The ROC curve demonstrated an exceptional AUC (exceeding 0.9), indicative of the high discriminatory capacity of the test in identifying abnormalities in ACR.

The calculation of the Youden Index facilitated the determination of an optimal cut-off point that ensures a balance between minimizing both false positives and false negatives. This cut-off point not only enhances the diagnostic accuracy of the test but also underscores its potential utility as a reliable screening tool in routine clinical practice.

Our study confirms a strong association and reliable correlation between ACR values obtained by Meditape UC-11A strips and the quantitative method. This supports the strip test as an effective and accessible screening tool for populations without a prior kidney disease diagnosis. Its ability to detect early signs of renal damage makes it a valuable resource for primary care, enabling timely interventions and improved outcomes.

Given that we are evaluating the Meditape UC-11A as a screening method, it is essential to examine the number of false negatives. We observe a false-negative rate of 2.21 %. All of these cases have values below 116 mg/g, and 70 % of them are below 50 mg/g. These findings may be explained by the tendency of the Meditape-UC11A to underestimate albumin levels while creatinine levels are often overestimated, especially at higher concentrations. However, careful monitoring in such cases should not be a problem in further control disease.

By determining the cut-off using the ROC curve, we have been able to reduce the number of false positives. However, even with this optimized cut-off, we still observed a 7.01 % rate of false positives. Further examination of these false positives revealed a notable trend: a considerable proportion of them exhibited ACR calculated by quantitative methods that were very close to the established cut-off point. Additionally, in cases where the urine reagent strip yielded a creatinine value of 10 mg/dL, the ACR result was consistently positive, contrasting with only 21.68 % confirmation using quantitative methods. This discrepancy suggests a potential limitation of the reagent strips in accurately measuring creatinine levels, particularly at lower concentrations.

Despite the inherent limitations of screening tests, the achieved low percentages of false positives and false negatives underscore their acceptable performance. These percentages are primarily influenced by the sensitivity of the technique, inherent to its screening nature. Consequently, the observed low false positives and false negatives rates are indicative of a successful outcome, aligning with our ultimate objective of reducing quantifications.

Our results indicate that the screening method using reagent strips has a sensitivity of 84.2 % and a specificity of 91.9 %, with PPV and NPV of 62.7 and 97.3 %, respectively. However, in previous studies that evaluated the same strip tests, the sensitivity, specificity, PPV and NPV for detecting albuminuria (UACR>30 mg/g) was 97–97.5 %, 44–67 %, 22–70.3 % and 97.1–99 % respectively [11], [12]. This variability may be attributed to differences in study populations, inclusion/exclusion criteria, methodologies used for ACR quantitative determination and differences in the definition of the cut-off.

Regarding laboratory implementation of a new working strategy, our study suggests that using reagent strips as a screening method followed by quantitative determination only in samples with positive results may be a cost-effective option. According to our analysis, with this strategy we can obviate the quantitative measurement of approximately 80 % of the requested urinary albumin test, resulting in significant economic savings.

Moreover, the semiquantitative ACR strip test offers several advantages over quantitative technology: it offers a rapid measurement process and takes advantage of existing laboratory protocols for urinalysis. Overall, it is useful not only to reduce economic costs but also staff and time saved can be redirected towards more critical tasks.

However, despite the encouraging findings, our study is not devoid of limitations. Future research endeavors should focus on validating these findings and determine the long-term clinical and economic impact of this screening strategy. Futhermore, the potential financial benefits we have identified may not translate universally to other countries or contexts, given that our laboratory operates within the Public Health Network.

In summary, our findings underscore the potential of Meditape UC-11A for ACR measurement as a valuable screening tool for detecting ACR abnormalities in PC patients. The robust AUC and the identified optimal cut-off support the clinical utility of this approach for early detection and management of renal function-related disorders. Implementing this strategy could not only aid in avoiding further unnecessary quantifications but also lead to considerable economic savings if applied on a daily basis.

## Supplementary Material

Supplementary Material
