# Supplementary material for: Evaluation of urine albumin-to-creatinine ratio analysis using strip test as a screening method for urinary albumin determination in primary care
Source: Adv Lab Med. 2025 May 1;6(3):345–50. doi: 10.1515/almed-2024-0068 (PMC12446912; doi:10.1515/almed-2024-0068)
Supplement: Supplementary file 1 — Supplementary Material [file j_almed-2024-0068_suppl_001.docx]

**Table S1: Sensitivity, Specificity, and Youden Index for different ACR analyzed by Meditape UC-11A strip (Sysmex, Kobe, Japan) thresholds, with the highest Youden index point highlighted.**

| **Threshold**  **(mg/g)** | **Sensitivity** | **Specificity** | **Youden Index** |
| --- | --- | --- | --- |
| 3,3333333 | 1 | 0 | 0 |
| 5 | 1 | 0,01998572 | 0,019985724 |
| 10 | 1 | 0,22055675 | 0,220556745 |
| 15 | 0,96902655 | 0,56316916 | 0,532195714 |
| 20 | 0,96017699 | 0,60242684 | 0,562603829 |
| 26,666667 | 0,84213274 | 0,91891577 | 0,763048518 |
| 30 | 0,83628319 | 0,92362598 | 0,759909167 |
| 35 | 0,71681416 | 0,93647395 | 0,653288106 |
| 40 | 0,71238938 | 0,93647395 | 0,648863328 |
| 50 | 0,67699115 | 0,94218415 | 0,619175305 |
| 60 | 0,65929204 | 0,94289793 | 0,602189965 |
| 75 | 0,53539823 | 0,94932191 | 0,484720143 |
| 80 | 0,45575221 | 0,95074946 | 0,406501677 |
| 100 | 0,35840708 | 0,95289079 | 0,311297872 |
| 105 | 0,31415929 | 0,99928622 | 0,313445516 |
| 150 | 0,30973451 | 0,99928622 | 0,309020737 |
| 160 | 0,19911504 | 0,99928622 | 0,198401268 |
| 300 | 0,14159292 | 1 | 0,14159292 |
| 800 | 0,02654867 | 1 | 0,026548673 |
| 1500 | 0,02212389 | 1 | 0,022123894 |
